# Mortality from Copper Smelter Emissions Circa 1967

**DOI:** 10.1289/ehp.10441

**Published:** 2007-09

**Authors:** Thomas J. Grahame

**Affiliations:** U.S. Department of Energy, Washington, DC, E-mail: thomas.grahame@hq.doe.gov

Pope et al. (2007) found lowered monthly mortality rates during a 1967–1968 copper smelter strike, coincident with and attributed to widespread reduced airborne sulfate levels. The authors cited three “intervention” studies associating particulate emissions reductions with mortality reductions as supportive. Evidence below suggests that mortality reductions in the study by Pope et al. and “interventions” are likely linked to reductions in particulate matter (PM) types known to be harmful: high levels of biologically active metals and partially burned carbon.

The first “intervention” study (Pope et al. 1992), examined PM–mortality associations over 4 years, encompassing closure of a Utah steel mill (sulfate not measured; sulfur dioxide levels were “low”). Mortality rates were 40% greater than expected when the mill was operating, suggesting toxicity of mill emissions. Strongest associations were with respiratory disease, then cardiovascular disease. Filter extracts when the mill was operating contained high levels of lead, copper, and zinc and were more toxic (Frampton et al. 1999).

Mattson and Guidotti (1980) found women living in communities near copper smelters (1968–1975) in Arizona experienced highly elevated relative risks (RRs) for acute respiratory disease mortality: averaged RR for all six mining towns (40,000 combined population) was 5.61. Later, Small et al. (1981) found levels of arsenic, Cu, and Zn elevated by factors up to 100,000 in Arizona smelter plumes. Lead levels in plumes were comparable with those of other metals. Thus, Pb, Cu, and Zn levels were elevated when either the steel mill or copper smelters were operating, and acute mortality (especially respiratory) was elevated simultaneously.

Mortality associations with blood Pb have recently been found at low levels of Pb (Menke et al. 2006). Blood Pb has a half-life of about 1 month, reflecting current exposure; associations may indicate both chronic and acute effects (Schober et al. 2006)—relevant information for copper smelter emissions.

The second “intervention” study (Hedley et al. 2002) found mortality rate reductions following a mandated 1990 reduction of sulfur in residual oil and diesel fuels in Hong Kong. Later, Hedley et al. (2006) found that ambient vanadium and nickel were reduced up to 90%, concomitantly with reductions of sulfur in residual oil. Mortality or inflammatory associations with ambient residual oil emissions but not secondary sulfate were previously found (Grahame and Hidy 2004; Janssen et al. 2002; Maciejczyk and Chen 2005).

The third “intervention” (Clancy et al. 2002) found that mortality rates declined after uncontrolled domestic burning of coal was banned in Dublin, Ireland. Wintertime black smoke levels declined from 80 μg/m^3^ before the ban to 20 μg/m^3^ afterward; sulfate was not measured. Given the toxicology of partly burned hydrocarbons, mortality reduction would be expected.

The three “supporting” studies do not provide evidence that widespread secondary sulfate reductions were related to mortality reductions during the interventions. Rather, high levels of specific metals, or of black smoke, appear to have health relevance.

Toxicology suggests secondary sulfates per se are unlikely to be harmful at ambient levels (Schlesinger and Cassee 2003). Resolving this inconsistency requires researching mechanisms by which secondary sulfate or precursors are necessary to create a toxic mixture at ambient levels; for example, how much do which metals increase in solubility due to such processes, and how much harm occurs that would not otherwise occur? Either soluble or insoluble metals common to steel mills and copper smelter emissions can be harmful at high doses (Ghio et al. 1999). Research suggestions are available (Grahame and Schlesinger (2007).
